# Analysis of Epileptic Discharges from Implanted Subdural Electrodes in Patients with Sturge-Weber Syndrome

**DOI:** 10.1371/journal.pone.0152992

**Published:** 2016-04-07

**Authors:** Yasushi Iimura, Hidenori Sugano, Madoka Nakajima, Takuma Higo, Hiroharu Suzuki, Hajime Nakanishi, Hajime Arai

**Affiliations:** Department of Neurosurgery, Epilepsy Center, Juntendo University, Tokyo, Japan; University of Pécs Medical School, HUNGARY

## Abstract

**Objective:**

Almost two-thirds of patients with Sturge-Weber syndrome (SWS) have epilepsy, and half of them require surgery for it. However, it is well known that scalp electroencephalography (EEG) does not demonstrate unequivocal epileptic discharges in patients with SWS. Therefore, we analyzed interictal and ictal discharges from intracranial subdural EEG recordings in patients treated surgically for SWS to elucidate epileptogenicity in this disorder.

**Methods:**

Five intractable epileptic patients with SWS who were implanted with subdural electrodes for presurgical evaluation were enrolled in this study. We examined the following seizure parameters: seizure onset zone (SOZ), propagation speed of seizure discharges, and seizure duration by visual inspection. Additionally, power spectrogram analysis on some frequency bands at SOZ was performed from 60 s before the visually detected seizure onset using the EEG Complex Demodulation Method (CDM).

**Results:**

We obtained 21 seizures from five patients for evaluation, and all seizures initiated from the cortex under the leptomeningeal angioma. Most of the patients presented with motionless staring and respiratory distress as seizure symptoms. The average seizure propagation speed and duration were 3.1 ± 3.6 cm/min and 19.4 ± 33.6 min, respectively. Significant power spectrogram changes at the SOZ were detected at 10–30 Hz from 15 s before seizure onset, and at 30–80 Hz from 5 s before seizure onset.

**Significance:**

In patients with SWS, seizures initiate from the cortex under the leptomeningeal angioma, and seizure propagation is slow and persists for a longer period. CDM indicated beta to low gamma-ranged seizure discharges starting from shortly before the visually detected seizure onset. Our ECoG findings indicate that ischemia is a principal mechanism underlying ictogenesis and epileptogenesis in SWS.

## Introduction

Sturge-Weber syndrome (SWS) is a congenital neurocutaneous disorder characterized by facial cutaneous nevus flammeus, leptomeningeal angioma, and glaucoma [1]. Clinical problems particular to neurology are migraine, hemiparesis, seizures, and mental retardation [2]. Maintenance of psychomotor development is the crucial therapeutic goal, and this requires prompt seizure control [3]. Seizures affect 75–90% of patients with SWS, and 50–60% cases are refractory to medical treatment [4,5]. Surgical interventions need to be considered for SWS patients with drug-resistant epilepsy. However, scalp electroencephalography (EEG) does not provide definitive information to determine the severity of epilepsy and the origin and propagation of epileptic discharges [6–8]. Therefore, the mechanisms of ictogenesis and epileptogenesis in SWS have not been clarified. To properly evaluate the severity and EEG findings, we have to understand the mechanism of epilepsy in SWS.

As previous reports have described, SWS is characterized by abnormal development of cortical veins [9] and venous drainage into the deep venous system [10].

The deficient outflow through cortical veins and dependence on the deep venous drainage system induces venous congestion, and consequently results in progressive cerebral ischemia [11–13]. Cerebral blood flow (CBF) studies using single photon emission computed tomography (SPECT) [14,15] and xenon-enhanced computed tomography [16] showed hypoperfusion of the brain under leptomeningeal angiomas in patients with SWS.

Progressive cortical atrophy on magnetic resonance imaging (MRI) [17] and calcification on computed tomography [18] are morphologic changes that can result from cerebral ischemia, which can induce seizures and compromise blood supply [19]. The mismatch between CBF supply and demand in the cerebral cortex may increase severity of epilepsy in patients with SWS. With brain development, the demand for cerebral blood flow(CBF) is increased. However ischemia caused by venous congestion in SWS patients is induced by the mismatch between CBF supply and demand, and in the natural time course of the disease, ischemia progresses. Based on these findings, we hypothesized that the ictogenesis and/or epileptogenesis in SWS are due to cerebral ischemia. In the present study, we tested our hypothesis from an electrophysiological viewpoint using electrocorticography (ECoG) data of patients with SWS who were implanted with subdural electrodes to detect seizure activity.

## Patients and Methods

### Clinical profiles

From 1986 to 2012, 94 patients with SWS were studied at the Juntendo University-Epilepsy Center in Tokyo, Japan. 59 of the 94 patients with SWS had drug-resistant epilepsy and 54 of them were surgically treated. Patients underwent a non-invasive diagnostic protocol for epilepsy that included seizure semiological evaluation, interictal scalp EEG, MRI, molecular imaging (SPECT and fluorodeoxyglucose-positron emission tomography [FDG-PET]), and psychomotor-development testing [20]. For presurgical evaluation in drug resistant epilepsy cases, video-EEG monitoring was indicated.

Five patients out of the 54, including a case we already reported [3], were those who had ECoG data collected with subdural electrodes. Other than these 5 patients, there was not investigated with intracranial EEG techniques. They presented with progressive psychomotor deterioration, undefined seizures, and no detectable epileptic discharges on scalp EEG. We speculated that they had several seizures, but their symptoms were unclear with undetectable EEG changes. To definitively evaluate their epileptic activity, we implanted subdural electrodes. Table 1 shows the patients’ demographic and clinical data. A neuroradiologist who was blinded to the patients’ backgrounds evaluated the imaging findings. Sufficiently sized craniotomies were designed to implant subdural grids under general anesthesia. Specially designed subdural grids (UNIQUE MEDICAL Co, Tokyo, Japan) were prepared, and the number of platinum electrodes (4-mm diameter and 10-mm distance) contacting the cerebral cortex ranged from 24 to 78, depending on the extent of each patient’s leptomeningeal angioma. The subdural electrodes covered almost the entire surface over the leptomeningeal angioma, as well as adjacent cortex. ECoG data were acquired using a Neuro Fax digital video EEG system (NIHON-KODEN, Tokyo, Japan) equipped with 128 channels and sampled data at 500 Hz with a 16-bit analog-to-digital converter. Medication was discontinued as necessary to allow seizure capture and ictal ECoG data analysis. We performed the disconnection epilepsy surgeries for all of the patients based on the ECoG data. Surgical procedures were as follows: the posterior quadrantectomy (PQT) [21] in three, the PQT with frontal lobe disconnection in one, and the hemispherotomy [22] in one. Four of the five patients were seizure-free after surgery, and one patient who underwent the PQT plus frontal lobe disconnection had reduced seizure frequency.

**Table 1:** Patient demographics and clinical data. F, frontal; P, parietal; T, temporal; O, occipital; L, left; R, right. This study included five SWS patients with progressive mental retardation aged from 1 to 9 years old. Most of the patients presented conspicuous seizure symptoms.

Case	Age/Sex	Age at seizure onset	Duration of epilepsy	Angioma location	Number of subdural electrodes	Number of electrodes covered lesional/ non-lesional	Number of seizures	Seizure symptoms	Average duration of the clinical seizures (min)	Type of resection	Seizure outcome (duration of post-surgical follow-up)
**1**	1.4y/M	2m	1.2y	R TOP	24	18/6	8	Facial twitching	13.6±10.1	PQT Frontal disconnection	CPS residual (7y)
**2**	1.8y/M	1.5m	1.6y	L FTOP	63	47/16	3	Motionless staring Respiratory distress	9.0±1.7	Hemispherotomy	Seizure free (8y)
**3**	2.5y/M	2y	0.5m	R TOP	42	30/12	6	Motionless staring Respiratory distress	1.6±0.5	PQT	Seizure free (6.5y)
**4**	4y/M	2.7y	1.3y	L FTP	71	57/14	2	Motionless staring Respiratory distress	18.5±19.1	PQT	Seizure free (7y)
**5**	9y/M	6m	8.5y	L TO	78	56/22	2	Motionless staring Respiratory distress	7.5±7.8	PQT	Seizure free (3y)

### Seizure discharges analysis

We recorded 21 seizures from the five patients during video ECoG monitoring (Table 1). All five patients presented complex partial seizures (CPS); four patients exhibited motionless staring and prolonged respiratory distress, and one patient manifested CPS with facial twitching. An investigator blinded to the patient’s clinical data selected the ictal discharges by visual inspection and evaluated the following analyses: 1) seizure onset zone (SOZ), 2) seizure propagation speed, 3) seizure duration, and 4) power spectrogram analysis on some frequency bands from 60 s before seizure onset.

Seizure onset was defined as the first sustained rhythmic change on ECoG that could be clearly visually distinguished from the background ECoG and interictal activity that led to a clear seizure discharge without a return to background activity. We confirmed that the location of the electrode contacted the cortical surface by taking a picture of the surgical field. The examiner selected three adjacent electrodes 10mm apart outside the SOZ to calculate the seizure propagation speed in cm/min, as the distance between the two electrodes and the duration from the first unequivocal changes of amplitude. We averaged the propagation speeds from these two adjacent distances for each seizure, and subsequently averaged the data from several seizures of each patient. Seizure duration was defined as seizure onset to termination on ECoG based on visual inspection. Seizure termination was defined as the point at which the rhythmic discharges ended or changed to low voltage background oscillation, and was averaged for each patient. We analyzed time power spectrograms on several frequency bands from 60s before seizure onset using EEG Complex Demodulation Method (CDM) analysis (NoruPro Light Systems, Miyuki Giken Inc. Tokyo, Japan). CDM analysis was performed using ECoG data with 75 μV sensitivity, a 0.03s time constant, and 200 Hz high-cut filter. We selected frequency bands between 1 and 80 Hz with a 20-ms temporal resolution, divided into 1–10, 10–15, 15–30, and 30–80 Hz. The power spectrogram measurement was carried out at the point of 60, 30, 15, 5, 4, 3, 2, and 1s before, as well as at seizure onset. Subsequently, we averaged the amplitude of each frequency band on the SOZ and compared them with those of the non-SOZ, which was defined as the electrodes that were at least 3 cm from the SOZ. We also analyzed the significance of differences for spectral power data on the SOZ at each time point. We selected 10–30 and 30–80Hz frequency bands for time points of 30, 15, 5s before, as well as at seizure onset.

### Statistical analysis

All statistical analyses were performed with IBM Statistical Package of the Social Sciences software version 22.0 (IBM Corp., Armonk, NY, USA) for Windows. The average power spectrograms of SOZ and non-SOZ electrodes were compared at each observation point using two-tailed, unpaired Student’s t-tests. These data demonstrated a normal distribution. We also compared the averaged power spectrogram data for each frequency band at the same time points. And we analyzed the significance of differences for spectral power data at each time point using one-way analysis of variance followed by Dunnett post hoc multiple comparison tests. We considered p <0.05 statistically significant.

All investigations included in the study were part of the routine clinical diagnostic protocol of SWS patients with intractable epilepsy and were performed under the care of the authors. Patient’s clinical informed consent for management (a standard written form in Japanese) included a statement that the data obtained through the protocol can be used for research purposes. No additional invasive or non-invasive procedures were performed for the purpose of this study, but only the already obtained electrophysiological records for clinical purpose were submitted to further analysis. This study has been approved by the ethical committee of our institution (No.14-158, Juntendo University). We used the below documents (S1, S2 and S3 Texts).

## Results

Interictal EEG findings showed depressed activity over part of the affected hemisphere. All 21 seizures initiated in cortical tissue beneath the leptomeningeal angiomas; seizure onset from the surrounding cortex was not observed. The SOZs were located in the central area of the leptomeningeal angiomas, and not in the periphery of the lesions, in all five patients. No developed cortical veins were observed within the areas with the leptomeningeal angioma, and there was some degree of cortical atrophy. All of the seizure discharges started as rhythmic activity within the delta to theta ranges.

**Fig 1:**
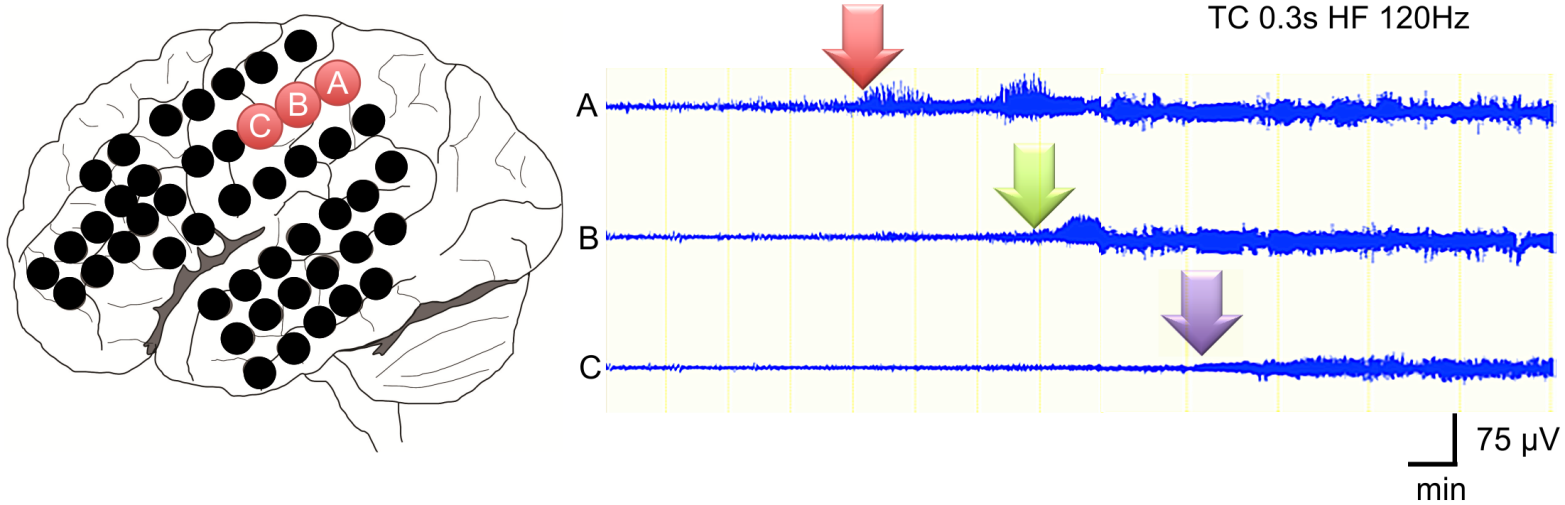
Seizure propagation speed analysis. Three adjacent electrodes 10-mm apart each at the SOZ were used to calculate the seizure propagation speed in cm/min. Details are presented in the methods section. This analysis shows an example of the propagation speed in a seizure in case 2. Seizure discharges gradually propagated from channel A to channels B and C at 3.7 cm/min.

All 21 seizures arose from single electrode. Therefore, we defined the SOZ as the first sustained rhythmic change on ECoG from a single electrode. The mean seizure propagation speed of all 21 seizures was 3.1 ± 3.6 cm/min., ranging from 0.3 to 10.0 cm/min (Table 2). Fig 1 shows an example of seizure propagation in a 20-month-old patient (Case 2). Seizure discharges gradually propagated from a channel to an adjacent one at 3.7 cm/min.

**Table 2:** Seizure propagation speed and duration.

	Number of seizures	Seizure propagation speed (cm/min)	Seizure duration (min)
**Case 1**	8	5.1 ± 4.5	36.4 ± 51.5
**Case 2**	3	2.9 ± 2.2	12.4 ± 2.8
**Case 3**	6	1.2 ± 0.2	2.6 ± 0.5
**Case 4**	2	0.8 ± 0.3	22.5 ± 21.9
**Case 5**	2	0.8 ± 0.3	9.0 ± 8.5
**Average**	-	3.1 ± 3.6	19.4 ± 33.6

The mean propagation speed of the seizures was 3.1 ± 3.6 cm/min, with a range of 0.3 to 10.0 cm/min. The mean duration of 21 seizures was 19.4 ± 33.6 min, with a range of 2.0 to 151 min. Older patients (cases 4, 5) tended to present a slower propagation speed.

**Fig 2:**
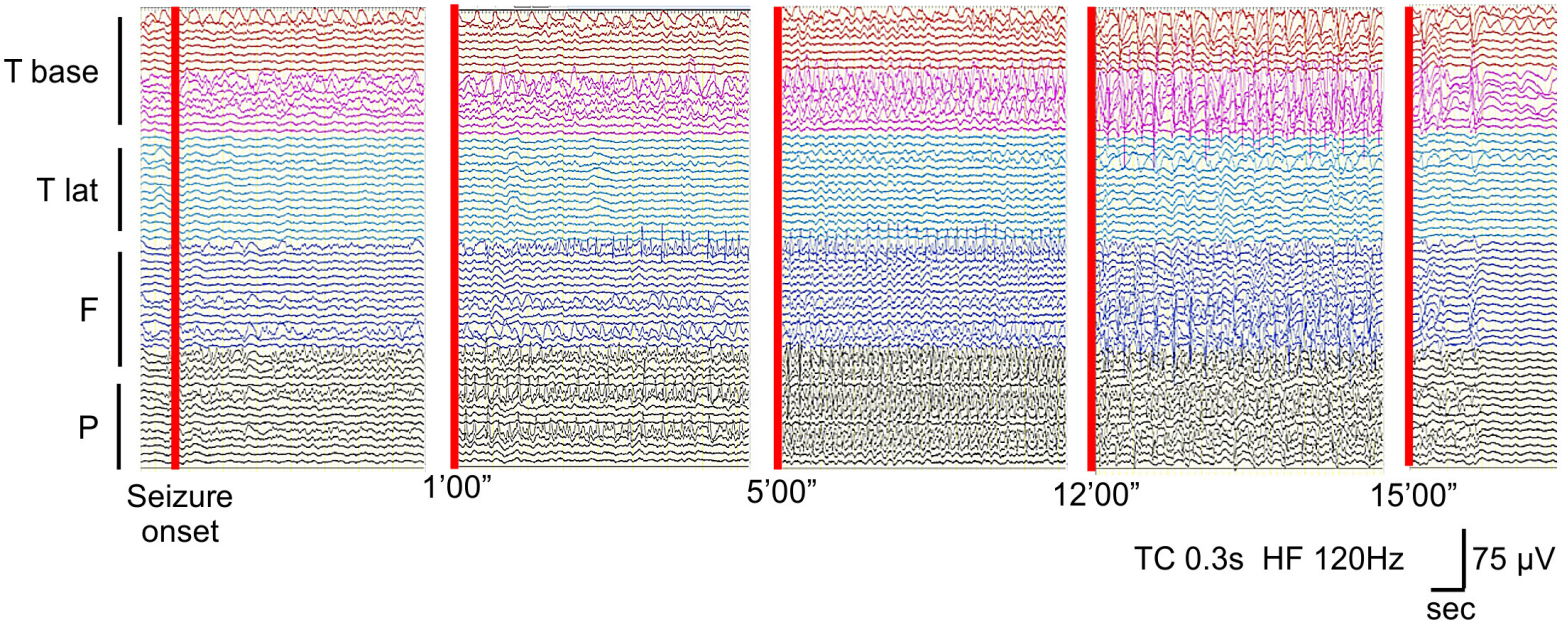
Seizure duration. This ECoG shows an example of a long-lasting seizure in case 2. Rhythmic seizure discharges gradually propagated from the base of temporal lobe to the frontal and parietal lobes over more than 15 min.

The mean seizure duration of all 21 seizures was 19.4 ± 33.6 min and ranged from 2.0 to 151 min (Table 2). Fig 2 shows the most obvious seizure in this series (Case 2), in which seizure discharges gradually propagated from the onset zone and lasted more than 15 min.

**Fig 3:**
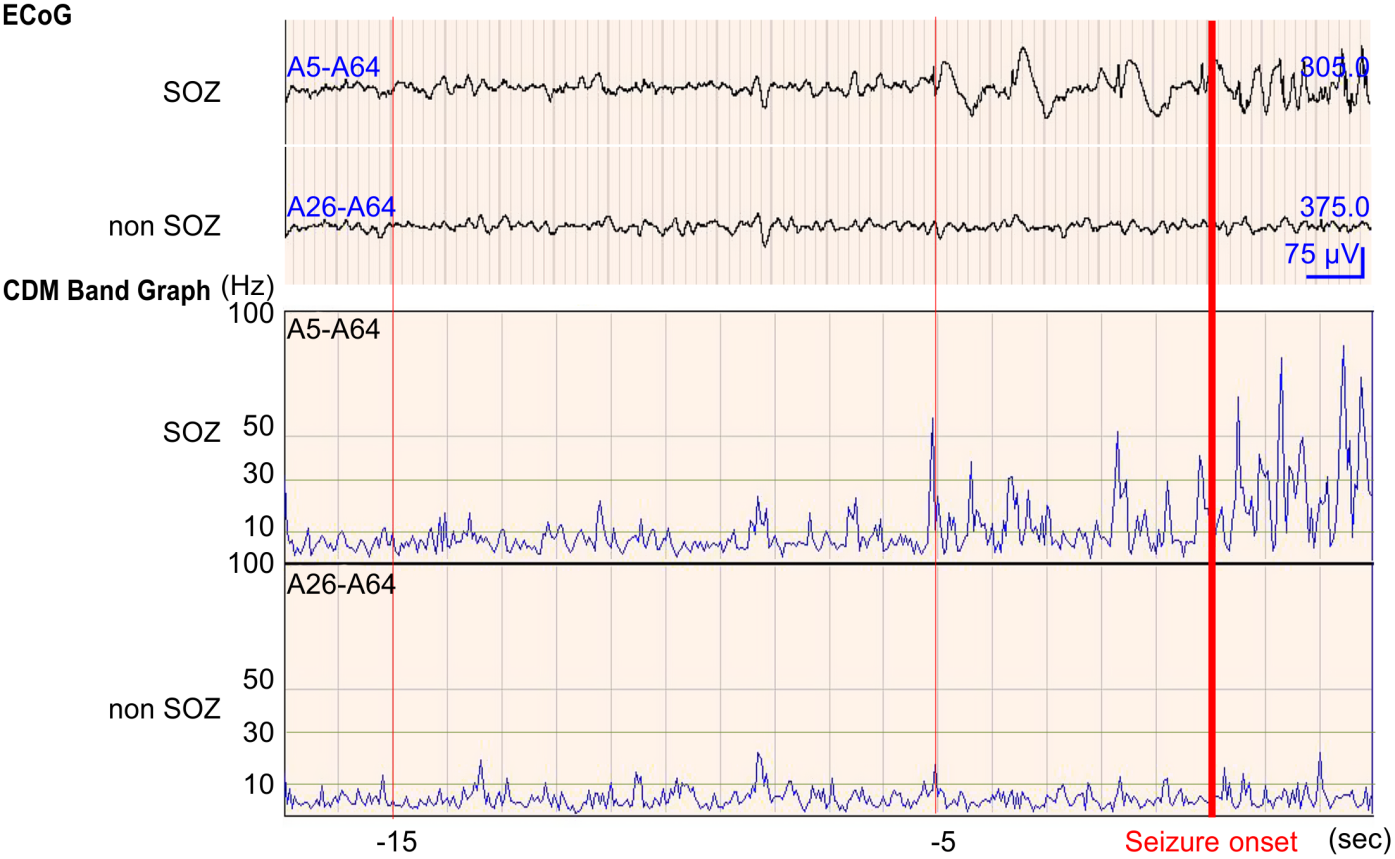
CDM band analysis associated with seizure onset on ECoG. Values for all x-axes are given in seconds. Values for all y-axes are given in Hz. The upper row showed ECoG at SOZ and non-SOZ electrodes, with the ECoG shown 20 s before and after seizure onset by visual inspection. The lower rows showed the CDM band graph between 0 and 100 Hz. Oscillations between 10 and 30 Hz were detected at SOZ from 15 s before seizure onset. Oscillations higher than 30 Hz were detected at SOZ from 5 s before seizure onset.

**Fig 4:**
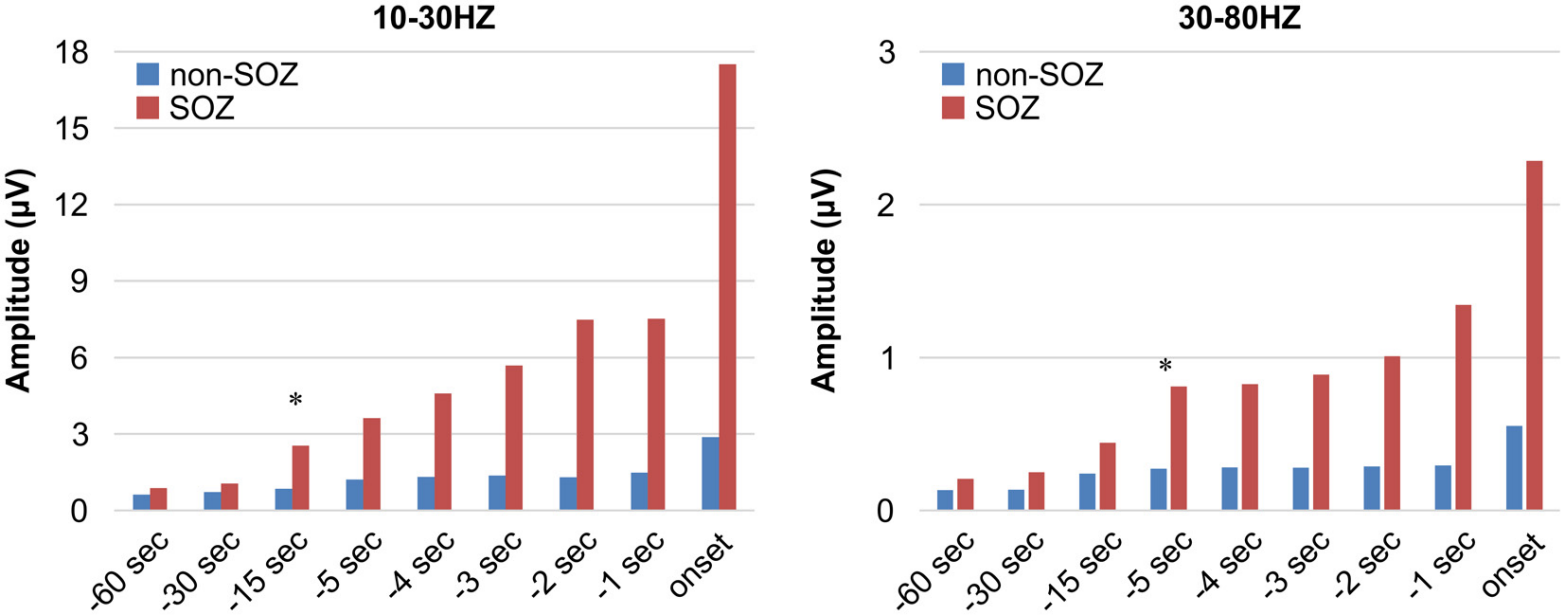
Statistical analysis of the frequency components in the SOZ and non-SOZ electrodes. A statistically significant difference in power spectrogram was detected at 10–30 Hz from 15 s before seizure onset and at 30–80 Hz from 5 s before seizure onset, respectively. * represents statistical significance (p < 0.05).

CDM band graph between 0 and 100 Hz and ECoG appearance for the same seizure are shown in Fig 3. The frequency of rhythmic seizure discharges on ECoG ranged from 3 to 7 Hz at the SOZ. However, oscillations between 30 and 80 Hz at the SOZ were detectable from 5 s before seizure onset through the CDM analysis. We averaged the power spectrograms at each frequency band and compared data from the SOZ and non-SOZ (Fig 4). A statistically significant difference in power spectrogram was observed between 10 and 30 Hz from 15 s before seizure onset, and a change between 30 and 80 Hz appeared from 5 s before the onset.

## Discussion

We hypothesized that the epileptogenesis of SWS is due to ischemia, and assessed this possibility in five patients using subdural electrode data. Although we were unable to accurately detect the seizures of these five patients on scalp video EEG, we determined the characteristics of their seizure symptoms, seizure frequency, and epileptic discharges using invasive EEG monitoring with subdural electrodes. Because SWS involves a thick leptomeningeal angioma covering the cerebral cortex, conventional scalp EEG can barely detect the low amplitude ictal and/or interictal epileptic discharges. Several studies have employed intracranial subdural electrodes and/or depth electrodes to obtain the electrophysiological findings for lesional cases, mainly in the malformation of cortical development (MCD) [23,24], and in non-lesional cases [25]. Previously two case reports were published [3,26] associated with intracranial EEG investigations. This study included a case we already reported [3]. According to Hata et al, a 19-month-old female infant with SWS was surgically treated under monitoring intraoperative ECoG [26]. They reported that seizures started posteriorly to the margin of the hemangiomatosis. These findings were different from the present study. We emphasized the analysis of multiple seizures and indicated that all of them arise from the cortex under the leptomeningeal angioma in our study. Only a few reports have described the use of an implanted subdural grid and strip electrodes for pediatric patients with intractable epilepsy attributable to ischemia [27,28].

Our findings suggest that these seizure waves propagate very slowly and persist over long periods. At these times, the seizures manifested with inconspicuous symptoms such as motionless staring and respiratory distress. These findings are similar to long-lasting epileptic discharges diagnosed as nonconvulsive status epilepticus (NCSE) [29]. The appearance of NCSE varies greatly on EEG and includes typical and atypical spike wave discharges, multiple or polyspike discharges, and rhythmic delta activity with intermixed spikes and evolution [29]. According to the literature, acute hypoxic-ischemic injury is the most common etiology of NCSE in pediatric patients [29]. Seizures in childhood, especially for neonates and infants, are likely to result from transient metabolic derangements such as hypoglycemia or hypocalcemia [29]. In immature brains with stroke, seizure is the only manifestation in 70% to 91% of patients and hemiparesis is uncommon only in 20% [30]. All seizures in our patients presented inconspicuous symptoms with prolonged rhythmic activity on ECoG with, and we can likely attribute these findings to NCSE. In the series of 23 patients with SWS, 12 (52%) had a history of status epilepticus, including non-convulsive status epilepticus [31] and we already reported one such case [3]. Furthermore, these epileptic discharges were derived from the venous congestive ischemic cortex underneath the leptomeningeal angioma. Therefore, the seizures were considered to have resulted from ischemia.

Similar to NCSE, the localized and long-lasting rhythmic epileptic discharges seemed to be periodic lateralized epileptiform discharges (PLEDs) [32]. PLEDs are characterized by prolonged EEG activity, and the most frequent underlying etiology is also ischemia [33]. A cerebral SPECT study presented reduction of regional cerebral blood flow (rCBF) at the timing of PLEDs on EEG [34]. Moreover, an animal study reported that seizure genesis occurs in both the infarct core and penumbra after stroke but PLEDs are generated in the penumbra [35]. Because the rCBF in the cortex under the leptomeningeal angioma in SWS is relatively low [36], the conditions in the lesions regarding blood perfusion are similar to those of the penumbra in ischemic stroke. From these previously reported findings, SWS can manifest as prolonged and rhythmic epileptic discharges similar to PLEDs.

From our time power spectrogram analysis at SOZ, beta oscillations were observed from 15 s before seizure onset, and low gamma oscillations appeared from 5 s before seizure onset. An electrophysiological study using ECoG after cerebral ischemia in an animal model showed that low gamma oscillations (30–70 Hz) and high gamma oscillations (80–120 Hz) occurred at the beginning of the initial spike [37]. Low gamma oscillations are predominantly found in the deeper cortical layers (LayerV), whereas high gamma oscillations (80–120 Hz) are more superficial (LayerII) [37]. Our data considered in the context of this animal study suggest that the initial ECoG change in the beta range triggers the seizure, and subsequent low gamma oscillation initiates and maintains the seizure discharges. The power of these oscillations was very low and could not be detected without CDM analysis. Because SWS is associated with long-lasting ischemia and results in progressive brain atrophy, epileptic discharges may not show increased amplitude as in an acute ischemic model. But we acknowledge the possibility that the identified preictal EEG changes might have been expression of already ongoing seizure activity, given that the changes were limited to the seizure-onset zone and that the actual time of seizure onset might have been missed. Thus, further EEG/ECoG studies are required for elucidating the ictogenic and epileptogenic mechanism in SWS, especially the meaning of the beta wave before seizure initiation.

Our ECoG study suggests that ischemia of the cortex under leptomeningeal angioma is a principal mechanism of ictogenesis and epileptogenesis in SWS. Seizure propagation speed tended to be slower in older patients (Table 2) than in infants in our study. This may mean that the affected brain in older children has been exposed to ischemia for a long time, and consequently has more advanced electrophysiological damage. The relationship between ischemia and seizure in SWS results in a vicious circle. Prolonged seizures even without conspicuous symptoms can expand ischemic damage and finally result in psychomotor deterioration. The only way to prevent expansion of damage in the brain and psychomotor deterioration in patients with SWS is to control the seizures using appropriate methods. To prevent progressive worsening of seizure control and psychological development, determination of surgical indications should concern targeting the ischemic lesions in the cortex under the leptomeningeal angioma.

## Conclusions

Our study indicates that seizures in patients with SWS are inconspicuous with slow propagation and long duration. Beta (10–30 Hz) to low gamma activities (30–80 Hz) was detected at the beginning of ictal onset in the cortex under leptomeningeal angioma. Our ECoG findings indicate that ischemia a principal mechanism of ictogenesis and epileptogenesis in SWS.

## Supporting Information

S1 Text (PDF)

S2 Text (PDF)

S3 Text (PDF)
