# Supplementary material for: Analysis of Epileptic Discharges from Implanted Subdural Electrodes in Patients with Sturge-Weber Syndrome
Source: PLoS One. 2016 Apr 7;11(4):e0152992. doi: 10.1371/journal.pone.0152992 (PMC4824532; doi:10.1371/journal.pone.0152992)
Supplement: S1 Text — (PDF) [file pone.0152992.s001.pdf]

<u>Notification letter of results of clinical trial examination</u>

Juntendo University Ethical Committee No.2014131
13/01/2015

Applicant: Assistant Professor Dr. Hidenori Sugano
Department of Neurosurgery

Juntendo University
The Head of the Medical School
Prof. Hajime Arai

CLINICAL TRIAL No. 14-158

<u>Official scientific title of the study: Rare intractable epilepsy syndrome registry</u>

<u>Principal investigator：　Assistant Professor　・　Hidenori Sugano</u>

Notice

Decision:	**Approval**
