# Supplementary material for: Analysis of Epileptic Discharges from Implanted Subdural Electrodes in Patients with Sturge-Weber Syndrome
Source: PLoS One. 2016 Apr 7;11(4):e0152992. doi: 10.1371/journal.pone.0152992 (PMC4824532; doi:10.1371/journal.pone.0152992)
Supplement: S2 Text — (PDF) [file pone.0152992.s002.pdf]

Official scientific title of the study: Rare intractable epilepsy syndromes registry and analysis

Title of the study (Brief title): RES-R

Objectives: We register and analyze the cases with rare intractable epilepsy (including various diseases with intractable epilepsy such as metabolic disorders or chromosomal anomalies) nationwide to obtain the approximate number of cases in Japan and estimate the mortality rate. We perform further analysis of the clinical and surgical data to elucidate the pathomorphology and pathophysiology of the rare types of epilepsy. We further perform a cross-sectional survey to reveal the actual conditions of patients and the relation between epilepsy duration and pathological status.

Key inclusion criteria:

Early myoclonic encephalopathy
Ohtahara syndrome
Epilepsy of infancy with migrating focal seizures
West syndrome
Dravet syndrome
Myoclonic status in nonprogressive encephalopathy
Epilepsy with myoclonic atonic seizures
Epilepsy with myoclonic absences
Lennox-Gastaut syndrome
Epileptic encephalopathy with continuous spike-and-wave during sleep
(Electrical Status Epilepticus during Slow Sleep)
Landau-Kleffner syndrome
Sturge-Weber syndrome.
Progressive myoclonus epilepsies
Mesial temporal lobe epilepsy with hippocampal sclerosis
Rasmussen syndrome
Gelastic seizures with hypothalamic hamartoma
Hemiconvulsion-hemiplegia-epilepsy syndrome
Aicardi syndrome
Angelman syndrome

Rett syndrome

PCDH19-related epilepsy in females

Ring 20 chromosome syndrome

Focal epilepsy NOS

Generalized epilepsy NOS

Undetermined epilepsy NOS
