# Supplementary material for: Analysis of Epileptic Discharges from Implanted Subdural Electrodes in Patients with Sturge-Weber Syndrome
Source: PLoS One. 2016 Apr 7;11(4):e0152992. doi: 10.1371/journal.pone.0152992 (PMC4824532; doi:10.1371/journal.pone.0152992)
Supplement: S3 Text — (PDF) [file pone.0152992.s003.pdf]

This is a translation of the additional to the clinical admission for epilepsy surgery informed consent, obtained for all patients in the study. In all cases the consents were signed by the parents of the patients, because of their age.

Informed Consent

1. I (we) agree the data obtained through the protocol to be used for research purposes. No additional invasive or non-invasive procedures will be performed for the purpose of a study, but only the already obtained electrophysiological records for clinical purposes will be submitted to further electronic off-line analysis.
2. We will use your EEG data for the analysis. All disclosed results of this study will be covered by all existing rules of protection of clinical personal data.
3. The performed analysis will not produce any harm or disadvantage to the patient and his relatives.
4. This informed consent can be withdrawn by the patient (relatives) at any time before, through or after the study.

If you agree to have the informed consent, please sign below

Date of agreement (YYYY/MM/DD): ______________

Patient's name: ______________________________

Representative's signature: ______________ (relation)

I provided the explanation about the informed consent.

Date of explanation (YYYY/MM/DD): 2015/10/21

Attending doctor: Yasushi Iimura
